# A New Series of Aryloxyacetic Acids Endowed with Multi-Target Activity towards Peroxisome Proliferator-Activated Receptors (PPARs), Fatty Acid Amide Hydrolase (FAAH), and Acetylcholinesterase (AChE)

**DOI:** 10.3390/molecules27030958

**Published:** 2022-01-31

**Authors:** Rosalba Leuci, Leonardo Brunetti, Antonio Laghezza, Luca Piemontese, Antonio Carrieri, Leonardo Pisani, Paolo Tortorella, Marco Catto, Fulvio Loiodice

**Affiliations:** Dipartimento di Farmacia-Scienze del Farmaco, Università degli Studi di Bari “Aldo Moro”, Via E. Orabona 4, 70125 Bari, Italy; rosalba.leuci@uniba.it (R.L.); leonardo.brunetti@uniba.it (L.B.); antonio.laghezza@uniba.it (A.L.); luca.piemontese@uniba.it (L.P.); antonio.carrieri@uniba.it (A.C.); leonardo.pisani@uniba.it (L.P.); paolo.tortorella@uniba.it (P.T.); marco.catto@uniba.it (M.C.)

**Keywords:** multi-target activity, PPAR agonists, enzymatic inhibition, Alzheimer’s disease

## Abstract

A new series of aryloxyacetic acids was prepared and tested as peroxisome proliferator-activated receptors (PPARs) agonists and fatty acid amide hydrolase (FAAH) inhibitors. Some compounds exhibited an interesting dual activity that has been recently proposed as a new potential therapeutic strategy for the treatment of Alzheimer’s disease (AD). AD is a multifactorial pathology, hence multi-target agents are currently one of the main lines of research for the therapy and prevention of this disease. Given that cholinesterases represent one of the most common targets of recent research, we decided to also evaluate the effects of our compounds on the inhibition of these specific enzymes. Interestingly, two of these compounds, **(*S*)**-**5** and **6**, showed moderate activity against acetylcholinesterase (AChE) and even some activity, although at high concentration, against Aβ peptide aggregation, thus demonstrating, in agreement with the preliminary dockings carried out on the different targets, the feasibility of a simultaneous multi-target activity towards PPARs, FAAH, and AChE. As far as we know, these are the first examples of molecules endowed with this pharmacological profile that might represent a promising line of research for the identification of novel candidates for the treatment of AD.

## 1. Introduction

Peroxisome proliferator-activated receptors (PPARs) control important metabolic functions in the body and are mainly implicated in lipid and glucose homeostasis, insulin sensitivity, and energetic metabolism. Over the years, PPARs have become a useful therapeutic target for the treatment of metabolic disorders comprising obesity, type 2 diabetes mellitus (T2DM), dyslipidemia, and hypertension [1,2]. The PPAR family comprises three different subtypes: α, β/δ and γ, whose expression and actions differ according to subtype, organ and tissue cell type [2].

PPARα or PPARγ agonist drugs, such as fibrates or thiazolidinediones (TZDs), have been widely employed for lipid and glycemic control [3,4]. On the other hand, no PPARδ agonists have been approved for clinical use [5]. However, recent studies have demonstrated that the full activation of these receptors is associated with unwanted effects [5,6]. To overcome these issues, the concept of selective PPAR modulators (SPPARMs) with a superior balance of efficacy and safety has been proposed [7,8]. SPPARMs are able to induce distinct agonistic and antagonistic responses depending on the cellular context and specific transcriptional signatures, shedding light onto a new possible path for the treatment of metabolic disorders.

It is worth noting that, in fact, there are no potent endogenous ligands of PPARs, and these receptors bind, with only moderate affinity, a variety of substances, most of which are of a lipidic nature. Thus, various signaling systems can interact with PPARs via shared mediators. The endocannabinoid system (ECS) is one of the most prominent among these signaling systems [9], and it is represented by the canonical cannabinoid receptors (CBRs) CB1 and CB2 and the endocannabinoid mediators (ECBs) arachidonoyl-ethanolamide (AEA, or anandamide) and 2-arachidonoyl-glycerol (2-AG). Other endogenous substances which, like AEA, belong to the family of *N*-acyl-ethanolamines (NAEs), can also interact with the ECS. The most prominent of these are oleoyl-ethanolamide (OEA) and palmitoyl-ethanolamide (PEA), which cannot be categorized as true endocannabinoids due to their lack of direct activity towards CBRs. Nevertheless, they exert their activity by competing with ECBs for fatty acid amide hydrolase (FAAH), which is one of their most important catabolic enzymes [9,10,11].

FAAH is a dimeric serine hydrolase localized on the membrane of the endoplasmic reticulum (ER), whose activity involves the rapid and complete hydrolysis of NAEs such as AEA, OEA and PEA. Its pharmacological inhibition logically determines increased concentrations of these compounds, causing FAAH to be most interesting as a possible therapeutic target, also due to the central role played by the upregulation of the ECS in many pathological processes such as pain, inflammation and cancer. Therefore, a pharmacological enhancement of the endocannabinoid tone could be useful for the treatment of these pathologies [12,13,14]. Importantly, it was proven that the inhibition of FAAH does not result in the psychoactive effects, namely sedation, hyperphagia and hypomotility, that are typically associated with phytocannabinoids like Δ^9^-tetrahydrocannabinol. FAAH inhibitors are also incapable of inducing tolerance, and their toxic effects are few and sporadic [12,13,14]. It is important to note that AEA, OEA, and PEA all act as PPARγ agonists, while only AEA is also capable of acting as a PPARα agonist [9,10,11]. Indeed, this overlap between the ECS and PPAR signaling makes the coupling of PPAR agonism with the enhancement of endocannabinoid tone (e.g., through FAAH inhibition) a very interesting avenue of research for novel pharmaceuticals, whose applications would range from the treatment of cancer [15], to neurodegenerative diseases [16], and alcohol withdrawal [17], other than merely for metabolic syndrome.

**Figure 1:**
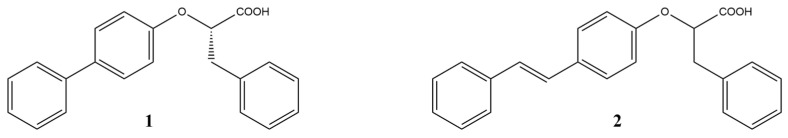
Chemical structure of aryloxyacetic acids **1** and **2**.

Recently, in order to obtain compounds capable of activating PPARs and inhibiting FAAH, we tested the inhibitory activity towards FAAH of a number of aryloxyacetic PPAR agonists, both known in the literature and synthesized in our laboratory. This biological evaluation was encouraged by the structural similarities between the aryloxyacetic class of PPAR agonists, whose activity has been widely studied in the past two decades [18,19,20,21], and the arylacetic class of cyclooxygenase (COX) inhibitors, which were recently shown to be moderately active on FAAH [22,23,24]. As a result, a few aryloxyacetic derivatives were found to be active as FAAH inhibitors and PPAR agonists [25]. In particular, the best multi-target activity was found for compound **2**, a trans-stilbenic analog of the well-known PPARα/γ dual agonist **1** [26] (Figure 1) which, on the contrary, showed a poor FAAH inhibitory activity.

**Figure 2:**
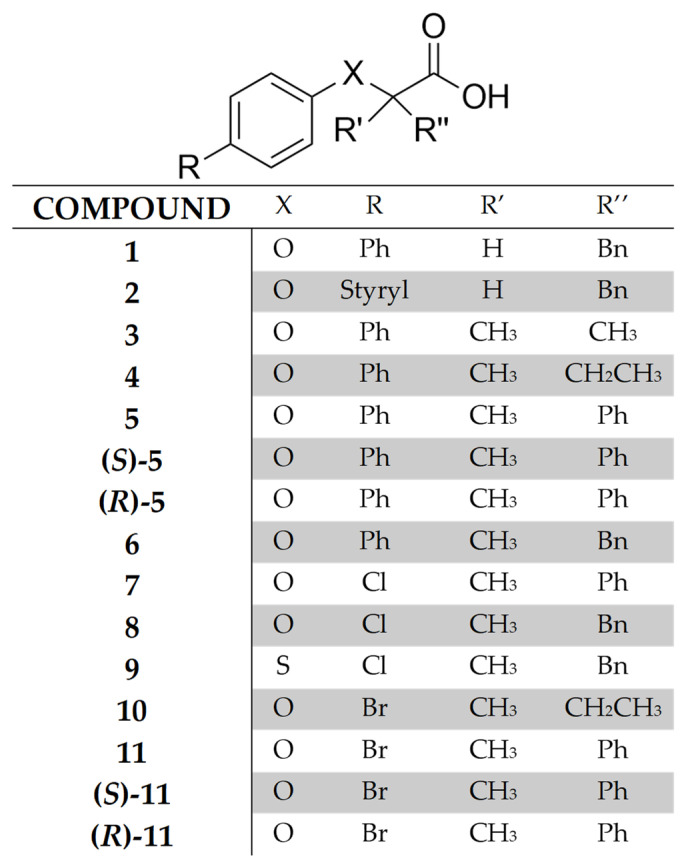
Chemical structure of the compounds studied in this work.

In order to investigate the structural modifications able to increase the multi-target activity of **1**, we decided to evaluate the effects resulting from the replacement of the distal benzene ring by a chlorine or bromine atom and/or from the introduction of a quaternary alpha carbon to the carboxylic group. The former modification could reveal the presence of a potentially beneficial halogen bond, whereas the latter was conceived because the presence of a sterically hindered quaternary carbon in bioactive small molecules could promote an element of conformational restriction that is shown to impart increased potency and metabolic stability [27]. However, before starting any synthetic efforts, we tested the adequacy of these chemical decorations by molecular dockings whose positive evidence prompted us to achieve the designed series of compounds (**3**–**11**, Figure 2).

Some of these new aryloxyacetic acids showed an appealing dual activity with a higher potency as FAAH inhibitors and PPARα/γ agonists compared to 2. Interestingly, this multi-target activity has been recently proposed as a new potential therapeutic strategy for the treatment of Alzheimer’s disease (AD) [16]. AD is a widespread pathology classified as a neurodegenerative disease consisting in a progressive loss of memory and cognitive functions [28,29,30]. Amyloid-β (Aβ) plaques that originated from the extraneuronal accumulation of Aβ peptides and intraneuronal aggregates of misfolded hyperphosphorylated tau protein are the main pathological hallmarks of AD [31,32]. Both of the aforementioned phenomena lead to neuronal loss and synapse dysfunction, especially in the cholinergic pathways in the brain, including the basal forebrain, hippocampus, and cerebral cortex, which are in charge of learning capability, memory, and other cognitive skills [33]. In particular, acetylcholinesterase (AChE) and butyrylcholinesterase (BuChE) seem to be responsible for a cholinergic activity deficit that leads to an overall loss of acetylcholine activity [34,35].

To date, the only treatments available for this disease are symptomatic, and no actual effective cure is available [36]. The general consensus on the nature of AD is that it is a multifactorial pathology, with both genetic and environmental components, and the dysregulation of many signaling and metabolic pathways seems to be involved in its pathogenesis. For this reason, multi-target agents are currently one of the main lines of research for the therapy and prevention of AD [37,38]. From this point of view, compounds with a dual activity as FAAH inhibitors and PPAR agonists could emulate and enhance the effects of endogenous *N*-acyl-ethanolamines by optimizing the existing synergies between the effects mediated by CBRs and those mediated by PPAR activation. In particular, a body of experimental evidence supports the idea that *N*-acyl-ethanolamines, acting via both canonical CB receptors and PPARs, can control the activity of various signaling pathways, like mitogen-activated protein kinase, nuclear factor-κB, Notch1 and Wnt/β-cat, through which they reduce neuroinflammation and hinder the formation of Aβ plaques and neurofibrillary tangles, resulting in an improvement of synaptic structure, synaptic plasticity and learning and memory deficits [39,40].

On the basis of these considerations, with the aim to investigate a possibly more extended activity profile of our new aryloxyacetic acid derivatives, we decided to also evaluate their effects on the inhibition of cholinesterases and Aβ peptide aggregation. In this case, docking studies were also preliminarily performed to test the possible interactions of our compounds with AChE, resulting in positive feedback. Interestingly, some of these compounds showed a moderate activity against AChE, demonstrating the feasibility of a simultaneous multi-target activity towards all four targets (PPARα, PPARγ, FAAH, AChE). As far as we know, these are the first examples of molecules endowed with this pharmacological profile, paving the way to a promising, yet unexplored, line of research for the identification of novel candidate drugs for the treatment of Alzheimer’s disease.

## 2. Results and Discussion

### 2.1. Dockings Studies

The molecular scaffold of **5** and **11** in both enantiomeric forms (Figure 2) was used as a three-dimensional scavenger to probe the capability of these aryloxyacetic acids to fit the binding sites of the aforementioned target proteins; to get a proper metric in the evaluation of the attained data, the reference compounds Wy-14,643, rosiglitazone, JZL195, and donepezil were also enrolled in this docking campaign.

From the data reported in Table 1 it might be perceived that, in each of the examined instances, these novel derivatives are capable of accomplishing favourable interactions with all the target counterparts. Indeed, not only the estimated free energy of binding but, more importantly, the ligand efficacy, which takes into account the contribution of each atom unit to the ligand-protein interaction process, ranks our molecules with scores similar to those of the reference compounds. In addition, docking also highlights very similar bindings for both enantiomers of **5** and **11**; in fact, these compounds, regardless of the relative stereochemistry, are able to accommodate the active gorge of PPARα, PPARγ, FAAH, and AChE demonstrating, at least in part, the previously postulated multi-target activity towards all four targets, as it might be perceived from the docking poses reported in Figure S1 (Supplementary Materials). These preliminary in silico data prompted us to carry out the planned synthesis of this new series of compounds.

### 2.2. Synthesis

**Scheme 1:**
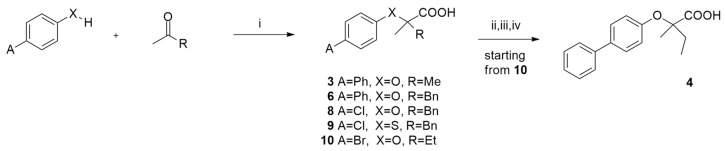
(**i**) KOH or NaOH, CHBr_3_ or CHCl_3_, room temperature, 16–36 h; (**ii**) MeOH, H_2_SO_4_, reflux, 4 h; (**iii**) Phenylboronic acid, Cs_2_CO_3_, Pd[(C_6_H_5_)_3_P]_4_, anhydrous toluene, reflux, 8 h; (**iv**) 2 N NaOH, THF, room temperature, overnight.

The aryloxyacetic acids **3**–**11** studied in this work are reported in Figure 2. Compounds **3**, **6** and **8**–**10** were prepared in a single step starting from the suitable commercial substituted phenol or thiophenol and the corresponding methyl ketone according to the Bargellini reaction. Chloroform or bromoform were used as reagents in the presence of KOH or NaOH solution as a base (Scheme 1). Compound **4** was prepared by reaction of the methyl ester of **10** with phenylboronic acid under Suzuki conditions and subsequent hydrolysis (Scheme 1).

**Scheme 2:**
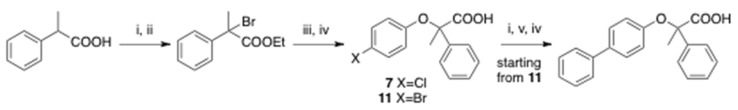
(**i**) EtOH, H_2_SO_4_, reflux, 4 h; (**ii**) *N*-bromosuccinimide, 33% HBr in acetic acid, CCl_4_, reflux, 24 h; (**iii**) 4-Cl or 4-Br-phenol, Na/EtOH or NaH/anhydrous DMF, 6–12 h; (**iv**) 2 N NaOH, THF, room temperature, overnight; (**v**) Phenylboronic acid, Cs_2_CO_3_, Pd[(C_6_H_5_)_3_P]_4_, anhydrous toluene, reflux, 8 h.

Scheme 2 describes the synthesis of compounds **5**, **7** and **11**. The ethyl ester of the commercially available 2-phenylpropionic acid was converted in the corresponding α-bromo-ester by reaction with NBS. Then, compounds **7** and **11** were obtained by nucleophilic substitution carried out with the suitable phenate and subsequent basic hydrolysis. Compound **5** was prepared starting from the ethyl ester of **11** which was reacted with phenylboronic acid under Suzuki conditions; the basic hydrolysis of the thus obtained intermediate afforded the target compound.

**Scheme 3:**
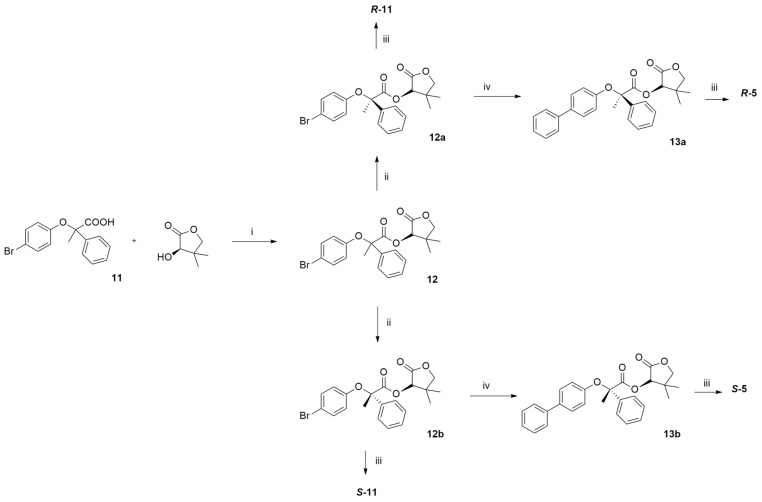
(**i**) DMAP, EDCI, CH_2_Cl_2_, room temperature, 24 h; (**ii**) silica gel column chromatography and recrystallisation from ethyl acetate/*n*-hexane 30:70; (**iii**) *i-*PrOH, 2.5 N NaOH, reflux, overnight; (**iv**) Phenylboronic acid, Cs_2_CO_3_, Pd[(C_6_H_5_)_3_P]_4_, anhydrous toluene, reflux, 8 h.

**Scheme 4:**
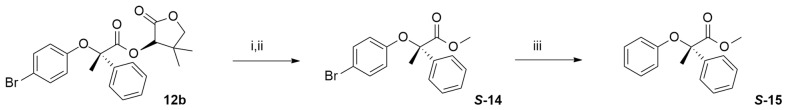
(**i**) 2.5 N NaOH, i-PrOH, 65 °C, 21 h; (**ii**) MeOH, H_2_SO_4_, reflux, 0.5 h; (**iii**) MeOH, 10% Pd/C, H_2_ 1.7 atm, 1 h.

**Figure 3:**
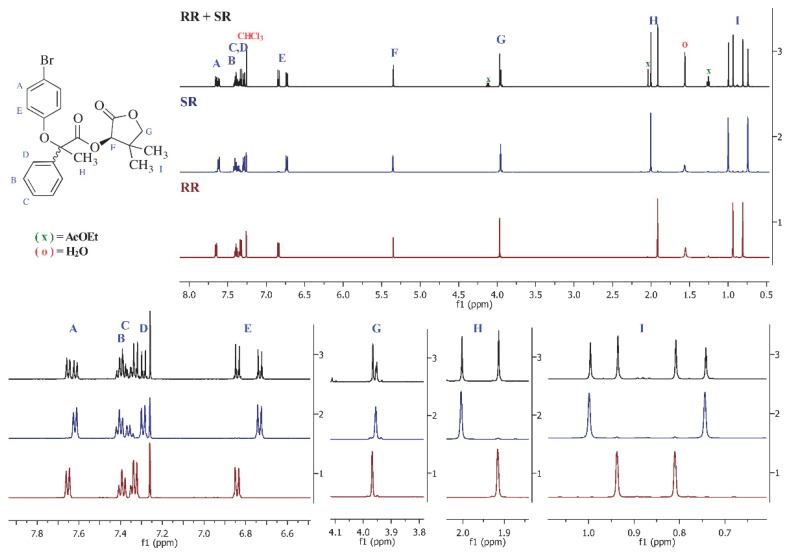
(**Top**) NMR spectra in CDCl_3_ (300 MHz) of the (*R*)-pantolactone diastereomeric esters **12a** and **12b**. RR + SR corresponds to the NMR spectrum of the mixture, SR and RR are the NMR spectra of the pure diastereomers. (**Bottom**) Magnification of the spectra above. Letters from A to I allow the identification of the protons as defined in the structure above. x: signals from AcOEt; o: signal from water.

The stereoisomers of compound **11** were resolved by column chromatography and fractional crystallization of the diastereomeric esters **12**, obtained through condensation of the racemic acid with (*R*)-pantolactone followed by hydrolysis (Scheme 3). On the other hand, the reaction of the diastereomerically pure esters **12a** and **12b** with phenylboronic acid under Suzuki conditions allowed for the obtaining of the compounds **(*R*)-5** and **(*S*)-5** by basic hydrolysis of intermediates **13a** and **13b**. The absolute configuration of the final acids was assigned through chemical correlation starting from **12b**, which was hydrolyzed, then esterified with methanol and finally dehalogenated by catalytic hydrogenation over 10% Pd/C to give compound (+)**−15** whose *S* configuration has been already reported in the literature [41] (Scheme 4). In this way, it was possible to assign the stereochemistry to both enantiomers of **5** and **11**. Their enantiomeric excess was >95%, as determined by NMR analysis of the diastereomeric (*R*)-pantolactone esters. In Figure 3 the NMR spectrum of the mixture of the two diastereoisomers is reported (*RR* + *SR*), whereas *SR* and *RR* are the NMR spectra of the pure diastereoisomers.

### 2.3. Biological Activity

Firstly, compounds **3**–**11** were evaluated in vitro for their agonist activity towards the human PPARα (hPPARα) and PPARγ (hPPARγ) subtypes by employing the GAL4-PPAR transactivation assay. For this purpose, GAL4-PPAR chimeric receptors were expressed in transiently transfected HepG2 cells according to a previously reported procedure [42]. In particular, the results obtained were compared with corresponding data for Wy-14,643 and rosiglitazone used as reference compounds in the PPARα and PPARγ transactivation assays, respectively. Maximum obtained fold induction with the reference agonist was defined as 100%. The activity of **3**−**11** was also compared with the lead compounds **1** and **2** (Table 2).

#### 2.3.1. PPARα Activity

The PPARα activity of racemates **3**–**11** was examined first. As shown in Table 2, almost all compounds behaved as full agonists. The introduction of a quaternary carbon atom in alpha to the carboxylic group did not produce relevant effects (**1** and **6** showed similar potency and efficacy), providing that a benzene ring was present. In fact, compounds **3**, **4** and **10** were the least active of the whole series. Moreover, the replacement of the distal benzene ring of **1** with a halogen atom also slightly reduced the activity as demonstrated by comparing the activity of compounds **5**, **7**, and **11** bearing the same substituents (methyl and phenyl) on the carbon in alpha to COOH. In this case, the bromine (compound **11**) resulted more beneficial than chlorine (compound **7**). Also, the presence of a sulphur atom bound to the quaternary carbon in place of an oxygen increased the activity, with **9** being twice as potent as **8**. With regard to stereochemistry, as previously reported for similar aryloxyacetic acids [18,42], *R* stereoisomers were inactive, whereas the *S* absolute configuration resulted in greater activity, as demonstrated from **(*S*)-5** and **(*S*)-11** which were the most potent derivatives of the series.

#### 2.3.2. PPARγ Activity

All compounds behaved as partial agonists except for **6** (full agonist) and **10,** which was completely inactive (Table 2). The activity on this receptor subtype was lower compared to PPARα, therefore, the differences were less evident in terms of potency and efficacy. However, the requisites for a higher activity were substantially similar: the presence of a benzene ring on the quaternary carbon and in the para position of the phenoxy group, and the *S* configuration of the stereoisomers. In fact, racemic acids **5** and **6** were the most potent of the series even though slightly less than **1**, whereas **(*S*)-5** and **(*S*)-11** ended up being about fivefold more potent than **(*R*)-5** and **(*R*)-11**, respectively. The most pronounced difference was shown from the sulphurated compound **9** that was twofold less potent and about fourfold less effective than the oxygenated derivative **8**. However, this behavior was in agreement with the previously reported results for arylthioacetic acids, whose greater lipophilic properties seem favorable for a higher activity on PPARα subtype [43].

On the whole, **(*S*)-5** and **6** displayed the most interesting pharmacological profile on both PPARα and PPARγ subtypes. Their potency and efficacy allow for the hypothesizing of the development of new dual agonists with a favorable and well-balanced activity.

#### 2.3.3. FAAH Inhibition Assay

Eight compounds out of thirteen were tested for FAAH inhibition by using the human recombinant enzyme, JZL-195, as a reference compound, and AMC-AA as a substrate. As shown in Table 2, the presence of a quaternary carbon was beneficial for inhibition activity; in fact, all compounds were more potent inhibitors compared to **1** and **2,** with IC_50_ ranging from 5.3 μM to 14.8 μM. On the contrary, the inhibition activity was not significantly affected from the presence of a phenyl on the quaternary carbon or in the para position of the phenoxy group. Surprisingly, even the stereochemistry was not critical given that both stereoisomers of **5** and **11** showed similar activity.

On the whole, the results reported above showed an appealing dual activity of some of these new aryloxyacetic acids as FAAH inhibitors and PPAR agonists. Interestingly, this multi-target activity has been recently proposed as a new potential therapeutic strategy for the treatment of AD [16]. For this reason, with the aim to investigate a possible more extended activity profile of these new compounds, we decided to also evaluate their effects on the inhibition of cholinesterases and Aβ peptide aggregation.

#### 2.3.4. Inhibition of Cholinesterases and Aβ Peptide Aggregation

The compounds assayed for FAAH inhibition were also tested as AChE and BuChE inhibitors via an in vitro assay, following a modification of Ellman’s spectrophotometric method [44] using donepezil as a reference compound. All derivatives were inactive on BuChE at the tested concentration, whereas, as shown in Table 3, the percentage of AChE inhibition at 10 μM ranged from 35 to 49%. The lack of a protonatable moiety, a key feature for an efficient interaction at the catalytic anion site of cholinesterases, may explain the low activity measured. Even though it could be irrelevant to explain the AChE/BuChE selectivity, it is possible that BuChE is not able to tolerate the presence of an acidic group in the molecules [45]. After all, as far as we know, only a few examples of carboxylic acids endowed with cholinesterase inhibition activity have been reported in the literature [45]. The restricted activity range towards AChE did not allow for the formulation of any comment about structure-activity relationships; however, as in FAAH inhibition, these preliminary experiments showed a low stereoselectivity of both enantiomers of **5** and **11** towards AChE. This is quite surprising for an enzyme like AChE, but it is reasonable to presume that the stereogenic center of these molecules is included in a region involved in interactions which do not present a constraining stereochemical demand.

As regards Aβ peptide aggregation, in vitro inhibition was assessed following a previously reported thioflavin T (ThT) fluorescence-based method involving the use of hexafluoroisopropanol (HFIP) as an aggregation enhancer [47]. As expected, all tested compounds showed only a low efficacy as aggregation inhibitors, even at 100 μM. In fact, previous studies have demonstrated the importance of the presence of (hetero)aromatic bi- or tricyclic systems to establish strong hydrophobic and electrostatic interactions with sequences of Aβ peptide being more prone to aggregating [48]. Also, the presence of a polar carboxylic group seems to impair the antiaggregating activity [45]. However, even the low efficacy of these compounds might be considered as a good starting point for the development of a suitable molecular scaffold endowed with the desired multi-target activity.

#### 2.3.5. ADME Properties

**Figure 4:**
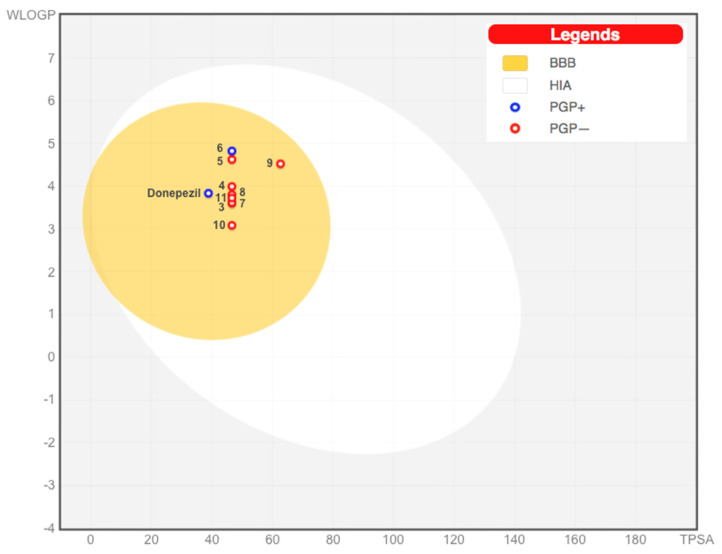
Pharmacokinetic profile of donepezil and compounds **3**–**11** according to BoiledEgg. BBB = blood-brain barrier permeation; HIA = human intestinal absorption; PGP+ = potential substrate of P−glycoprotein, PGP− = non potential substrate of P−glycoprotein.

The drug-likeness features of all compounds were further predicted by the Brain (or IntestinaL Estimate) permeation method [49], which suggests that the total of the studied compounds is well absorbed in the gastrointestinal tract as well as pass the blood brain barrier according to their lipophilicity and polarity, measured by WLOGP and TPSA respectively, that indeed largely resemble donepezil (Figure 4). Furthermore, except for **6**, they also might not be substrates of P-glycoprotein.

## 3. Materials and Methods

### 3.1. Chemical Methods

Reagents and solvents were purchased from common suppliers and were used without any further purification. Column chromatography was conducted using Geduran silica gel 60 A° (63–200 µm) as a stationary phase. Mass spectrometry was conducted on a HP MS 6890-5973 MSD spectrometer, electron impact 70 eV, equipped with a HP ChemStation or with an Agilent LC–MS 1100 Series LC–MSD Trap System VL spectrometer, and electrospray ionization (ESI). ^1^H-NMR spectra were recorded in the suitable deuterated solvent on Varian Mercury 300 NMR or Agilent VNMRS500 spectrometers. Chemical shifts (δ) are reported as parts per million (ppm), while the coupling constants (J) are measured in Hertz (Hz). Melting points are uncorrected and were measured in open capillaries on a Gallenkamp electrothermal apparatus (Fisons Erba Science Ltd., Guildford, UK). Optical rotations were measured with a PerkinElmer 341 polarimeter at room temperature (20 °C): concentrations are expressed as grams per 100 mL. The enantiomeric excesses of the final acids **(*R*)-5**, **(*S*)-5**, **(*R*)-11**, and **(*S*)-11** were >98% as determined by NMR analysis of the diastereomeric pantolactones **12a** and **12b**. Exact mass analyses or microanalyses of the tested compounds were within ±0.4% of the theoretical values except for compound **8,** whose microanalysis afforded a percentage of carbon and hydrogen higher than ±0.4% of the theoretical values (about 1%).

#### 3.1.1. Preparation of (4-Phenylphenoxy)-2-methylpropanoic Acid (**3**)

NaOH (powder, 20 mmol, 10 eq) was added to a solution of 4-phenyl-phenol (2 mmol, 1 eq) in acetone (5 mL). After 0.5 h at room temperature, CHCl_3_ (5.6 mmol, 2.8 eq) was added dropwise, during 30 min, to the reaction mixture. The resulting solution was refluxed for 3 h and stirred at room temperature overnight after which the organic solvent was distilled off and the residue added with distilled water. The aqueous phase was carefully acidified with 6 N HCl and extracted with ethyl acetate. The collected organic phase was washed with brine, dried over Na_2_SO_4_, filtered, and evaporated to dryness affording a brown oily residue, which was dissolved in ethyl acetate and extracted five times with a NaHCO_3_ saturated solution. The aqueous phase was carefully acidified with 6 N HCl and extracted four times with ethyl acetate. The collected organic layer was dried over Na_2_SO_4_, filtered, and evaporated to dryness to give a solid residue, which was recrystallized from dichloromethane to give the title compound as a solid; yield = 46%; m.p. = 172–173 °C; ^1^H-NMR (500 MHz, CDCl_3_) δ (ppm): 1.65 (s, 6H, CH_3_), 7.00–7.02, 7.31–7.35, 7.41–7.44 and 7.50–7.55 (m, 9H, aromatics); ESI-HRMS (C_16_H_16_O_3_) *m/z* (%) negative [M-H]^−^: calculated: 255.1027, found: 255.1024.

#### 3.1.2. Preparation of (4-Phenylphenoxy)-2-methyl-3-phenylpropanoic Acid (**6**)

NaOH (powder, 10 mmol, 5 eq) and phenylacetone (0.53 mL, 3 eq) were added to a solution of 4-phenyl-phenol (2 mmol, 1 eq) in THF (15 mL) at 0 °C. After 0.5 h at room temperature, CHCl_3_ (0.8 mL, 5 eq) was added dropwise, during 1 h, to the reaction mixture. The resulting solution was stirred at room temperature overnight, after which the organic solvent was distilled off and the dark solid residue was added with distilled water and washed with diethyl ether. The aqueous phase was carefully acidified with 2 N HCl (pH = 1) and extracted with ethyl acetate. The collected organic phase was extracted with an NaHCO_3_ saturated solution. The aqueous phase was carefully acidified with 6 N HCl and extracted four times with ethyl acetate, washed with brine, dried over Na_2_SO_4_, filtered, and evaporated to dryness affording a white solid which was recrystallized from chloroform/*n*-hexane; yield = 35%; m.p. = 149–150 °C; ^1^H-NMR (500 MHz, CD_3_OD) δ (ppm): 1.40 (s, 3H, CH_3_), 3.19 and 3.35 (2d, 2H, CH_2_, J = 13.2 Hz), 6.94–6.97, 7.22–7.31, 7.36–7.40 and 7.48–7.56 (m, 14H, aromatics); ESI-HRMS (C_22_H_20_O_3_) *m/z* (%) negative [M-H]^−^: calculated: 331.1340, found: 331.1335.

#### 3.1.3. Preparation of (4-Chlorophenoxy)-2-methyl-3-phenylpropanoic Acid (**8**)

KOH (powder, 12 mmol, 6 eq) was added to a solution of 4-chloro-phenol (2 mmol, 1 eq) in phenylacetone (3.7 mL, 14 eq). After 0.5 h at room temperature, CHBr_3_ (0.4 mL, 2.2 eq) was added dropwise, during 1.5 h, to the reaction mixture. The resulting solution was stirred at room temperature for 36 h after which the organic solvent was distilled off and the brown oily residue was added with distilled water. The aqueous phase was carefully acidified with 6 N HCl (pH = 1) and extracted with ethyl acetate. The collected organic phase was washed with NH_4_Cl saturated solution, dried over Na_2_SO_4_, filtered, and evaporated to dryness affording a brown oily residue, which was chromatographed on a silica gel column (dichloromethane/2-propanol 99:1 as eluent) affording the desired acid as a yellow oil. NaHCO_3_ (powder, 1 eq) was added to a solution of this acid (1 eq) in EtOH 95°. The mixture was stirred at room temperature for 20 h. The solvent was then evaporated to dryness and the residue was recrystallized from dichloromethane to give the title compound as a white solid; yield = 15%; m.p.= 225 °C dec. ^1^H-NMR (300 MHz, DMSO-d6) δ (ppm): 1.13 (s, 3H, CH_3_), 2.99 and 3.19 (2d, 2H, CH_2_, J = 13.5 Hz), 6.81–6.85 and 7.14–7.36 (m, 9H, aromatics); GS-MS (methyl ester with diazomethane): 306 (6) [M]^+^, 304 (17) [M]^+^, 245 (14), 177 (20), 121 (100), 91 (37); anal.: calcd for C_16_H_14_ClO_3_Na·2H_2_O: C 55.10 %, H 5.20 %, found: C 56.10 %, H 4.22 %.

#### 3.1.4. Preparation of (4-Chloro-phenylsulfanyl)-2-methyl-3-phenylpropanoic Acid (**9**)

NaOH (powder, 20 mmol, 10 eq) and phenylacetone (2.65 mL, 10 eq) were added to a solution of 4-chloro-thiophenol (2 mmol, 1 eq) in THF (10 mL) at 0 °C. After 0.5 h at room temperature, CHCl_3_ (0.63 mL, 4 eq) was added dropwise, during 0.5 h, to the reaction mixture. The resulting solution was stirred at room temperature overnight, after which the organic solvent was distilled off and the orange oily residue was added with distilled water and extracted with ethyl acetate. The collected organic phase was washed with 2 N HCl and washed with brine, dried over Na_2_SO_4_, filtered, and evaporated to dryness affording a residue which was recrystallized from *n*-hexane to give the title compound as a white solid; yield = 32%; m.p. = 118–120 °C; ^1^H-NMR (300 MHz, CDCl_3_) δ (ppm): 1.34 (s, 3H, CH_3_), 2.92 and 3.40 (2d, 2H, CH_2_, J = 13.6), 7.21–7.48 (m, 9H, aromatics); GS-MS (methyl ester with diazomethane): 320 (41) [M]^+^, 261 (9), 229 (100), 121 (74), 91 (37); anal.: calcd for C_16_H_15_ClO_2_S: C 62.64 %, H 4.93 %, found: C 63.15 %, H 4.93 %.

#### 3.1.5. Preparation of (4-Bromophenoxy)-2-methylbutanoic Acid (**10**)

KOH (powder, 12 mmol, 6 eq) was added to a solution of 4-bromo-phenol (2 mmol, 1 eq) in 2-butanone (6 mL). After 0.5 h at room temperature, CHBr_3_ (0.4 mL, 2.2 eq) was added dropwise, during 1.5 h, to the reaction mixture. The resulting solution was stirred at room temperature overnight, after which the organic solvent was distilled off and the oily residue was added with distilled water and washed with CHCl_3_. The aqueous phase was carefully acidified with 6 N HCl (pH = 1) and extracted with chloroform. The collected organic phase was washed with brine, dried over Na_2_SO_4_, filtered, and evaporated to dryness, affording a brown residue which was recrystallized from *n*-hexane to give the title compound as a yellow solid; yield = 65%; m.p. = 100–101 °C; ^1^H-NMR (300 MHz, CDCl_3_) δ (ppm): 1.03 (t, 3H, CH_3,_ J = 7.6), 1.49 (s, 3H, CH_3_), 1.89–2.07 (m, 2H, CH_2_), 6.81–6.86 and 7.36–7.41 (m, 4H, aromatics); GC-MS (methyl ester with diazomethane) *m/z* (%): 288 (19) [M+2]^+^, 286 (19) [M]^+^, 229 (22), 227 (22), 174 (100). ESI-HRMS (C_11_H_13_BrO_3_) *m/z* (%) negative [M-H]^−^: calculated: 270.9975, found: 270.9975.

#### 3.1.6. Preparation of Methyl (4-Bromophenoxy)-2-methylbutanoate

Compound **10** (2 mmol) was dissolved in methanol (7 mL) and added with a catalytic amount of H_2_SO_4_, and the resulting mixture was refluxed for 4 h. Then, the solvent was concentrated under reduced pressure and the resulting oil was dissolved in ethyl acetate and washed with sodium bicarbonate aqueous solution and brine. The organic portion was dried over anhydrous Na_2_SO_4_, filtered, and concentrated to dryness, affording the title compound as a yellow oil, yield 83%; ^1^H-NMR (300 MHz, CDCl_3_): δ 1.32 (t, 3H, CH_2_CH_3_, J = 6.4 Hz), 1.81 (s, 3H, OCCH_3_), 2.31 (q, 2H, CH_2_CH_3_, J = 6.4 Hz), 7.01–7.34 and 7.44–7.82 (m, 4H, aromatics), 9.51 (broad singlet, 1H, COOH, D_2_O exchanged); GC-MS *m/z* (%): 288 (19) [M+2]^+^, 286 (19) [M]^+^, 229 (22), 227 (22), 174 (100).

#### 3.1.7. Synthesis of Methyl (4-Phenylphenoxy)-2-methylbutanoate

Phenylboronic acid (4 mmol, 2 eq) and Cs_2_CO_3_ (3 mmol, 1.5 eq) were added, under a N_2_ atmosphere, to a stirred solution of methyl (4-bromophenoxy)-2-methylbutanoate (2 mmol, 1 eq) in a mixture of toluene and water (21 mL, 20:1); after 1 h at RT, Pd(PPh_3_)_4_ (0.06 mmol, 0.03 eq) was added. The reaction mixture was refluxed overnight and then quenched with 2 N HCl and ethyl acetate (10 mL, 1:1). The suspension was filtered through a Celite pad to remove the catalyst, and the filtrate was concentrated to dryness to give an oil. The crude was dissolved in water and extracted with ethyl acetate three times. The collected organic portions were washed with brine, dried over anhydrous Na_2_SO_4_, filtered and concentrated to dryness, obtaining a brown solid in 62% yield. ^1^H-NMR (300 MHz, CDCl_3_) δ (ppm): 1.02 (t, 3H, CH_3,_ J = 7.5), 1.57 (s, 3H, CH_3_), 1.92–2.13 (m, 2H, CH_2_), 3.80 (s, 3H, CH_3_) 6.87–6.96 (m, 2H, aromatics), 7.24–7.61 (m, 7H, aromatics); GC-MS *m/z* (%): 284 (13) [M]^+^, 170 (100).

#### 3.1.8. Synthesis of Methyl (4-Phenylphenoxy)-2-methylbutanoic Acid (**4**)

2 N NaOH (26 mmol in 13 mL of H_2_O, 13 eq) was added to a solution of methyl (4-phenylphenoxy)-2-methylbutanoate (2 mmol, 1 eq) in THF (13 mL). The resulting mixture was stirred overnight at room temperature. Next, the organic solvent was removed under reduced pressure, the aqueous residue was acidified with 6 N HCl and then extracted with ethyl acetate. The collected organic portions were washed with brine, dried over anhydrous Na_2_SO_4_, filtered and concentrated to dryness. The solid residue was recrystallized from CHCl_3_/*n*-hexane affording the title compound as a white solid, yield 32%. m.p. = 140–141 °C; ^1^H-NMR (300 MHz, CDCl_3_) δ (ppm): 1.08 (t, 3H, CH_3,_ J = 7.6), 1.55 (s, 3H, CH_3_), 1.84–2.12 (m, 2H, CH_2_), 7.01–7.04 and 7.30–7.56 (m, 9H, aromatics). ESI-HRMS (C_17_H_18_O_3_) *m/z* (%) negative [M-H]^−^: calculated: 269.1183, found: 269.1184.

#### 3.1.9. Preparation of Ethyl 2-Phenylpropanoate

2-Phenylpropanoic acid (2 mmol) was dissolved in ethanol (5 mL) and added with a catalytic amount of H_2_SO_4_, and the resulting mixture was refluxed for 4 h. Then, the solvent was concentrated under reduced pressure and the resulting oil was dissolved in ethyl acetate and washed with sodium bicarbonate aqueous solution and brine. The organic portion was dried over anhydrous Na_2_SO_4_, filtered, and concentrated to dryness, affording the title compound as a yellow oil, yield 84%; ^1^H-NMR (500 MHz, CDCl_3_) δ (ppm): 1.15 (t, 3H, CH_2_CH_3_, J = 7.1), 1.45 (d, 3H, CHCH_3_, J = 7.2), 3.66 (q, 1H, CH, J = 7.2), 4.02–4.13 (m, 2H, CH_2_), 7.17–7.30 (m, 5H, aromatics); GC-MS *m/z* (%): 178 (17) [M]^+^, 105 (100).

#### 3.1.10. Preparation of Ethyl 2-Bromo-2-phenylpropanoate

Ethyl 2-phenylpropanoate (2 mmol) was dissolved in CCl_4_ (0.4 mL) and mixed with *N*-bromo-succinimide (6 mmol) in a 1:2.3 stoichiometric ratio. Hydrobromic acid (33% in acetic acid) was then added in a catalytic amount. The mixture was stirred and heated under reflux for 24 h, then cooled to room temperature and filtered using a Gooch funnel. The resulting solution was washed with brine, dried over anhydrous Na_2_SO_4_, filtered, and concentrated to dryness, affording the title compound as an amber oil, yield 99%; ^1^H-NMR (300 MHz, CDCl_3_) δ (ppm): 1.27 (t, 3H, CH_3_, J = 7.1 Hz), 2.29 (s, 3H, CH_3_), 4.27 (q, 2H, CH_2_, J = 7.1 Hz), 7.26–7.38 and 7.54–7.58 (m, 5H, aromatics). GC-MS *m/z* (%): 256 (1) [M]^+^, 185 (27), 183 (28), 177 (100), 103 (55).

#### 3.1.11. Preparation of Ethyl 2-(4-Bromophenoxy)-2-phenylpropanoate

NaH (4 mmol, 2 eq) was suspended in anhydrous DMF (6 mL), then 4-bromo-phenol (4 mmol, 2 eq) and, after 30′, ethyl 2-bromo-2-phenylpropanoate (2 mmol, 1 eq) were dissolved in anhydrous DMF (3.5 mL) and added dropwise at 0 °C. The reaction was stirred under inert atmosphere at room temperature for 6 h. Then, DMF was distilled off and the residue dissolved in ethyl acetate. The organic solution was washed with an aqueous solution of NH_4_Cl, 0.5 N NaOH and brine, dried over anhydrous Na_2_SO_4_, filtered and evaporated under reduced pressure. The resulting crude was purified by chromatography column (eluent *n*-hexane/ethyl acetate 8:2), to give a brown oil, yield 81%; ^1^H-NMR (300 MHz, CDCl_3_) δ (ppm): 1.16 (t, 3H, CH_3_, J = 7.1 Hz), 1.88 (s, 3H, CH_3_), 4.19 (q, 2H, CH_2_, J = 7.1 Hz), 6.70–6.75, 7.26–7.40 and 7.56–7.60 (m, 9H, aromatics). GC-MS *m/z* (%): 350 (2) [M+2]^+^, 348 (2) [M]^+^, 277 (17), 275 (18), 177 (100), 103 (94).

#### 3.1.12. Preparation of Ethyl 2-(4-Chlorophenoxy)-2-phenylpropanoate

Na (4 mmol, 2 eq) was dissolved in absolute ethanol (6 mL), then 4-chloro-phenol (4.4 mmol, 2.2 eq) and, after 3 h at room temperature, ethyl 2-bromo-2-phenylpropanoate (2 mmol, 1 eq) were dissolved in absolute ethanol (4 mL), and added dropwise. The reaction was stirred under reflux for 12 h. Then, the solvent was distilled off and the residue dissolved in ethyl acetate. The organic solution was washed with 2 N HCl, brine, 2 N NaOH and brine, dried over anhydrous Na_2_SO_4_, filtered and evaporated under reduced pressure to give a yellow oil, yield 32%. ^1^H-NMR (300 MHz, CDCl_3_) δ (ppm): 1.20–1.30 (m, 3H, CH_3_), 1.79 (s, 3H, CH_3_), 4.14–4.25 (m, 2H, CH_2_), 6.72–6.78 (m, 2H, aromatics), 7.12–7.20 (m, 2H, aromatics), 7.28–7.62 (m, 5H, aromatics); GC-MS *m/z* (%): 304 (4) [M]^+^, 233 (10), 231 (29), 177 (100), 149 (35), 131 (37), 77 (28).

#### 3.1.13. Synthesis of Ethyl (4-Phenylphenoxy)-2-phenylpropanoate

Phenylboronic acid (4 mmol, 2 eq) and Cs_2_CO_3_ (3 mmol, 1.5 eq) were added, under a N_2_ atmosphere, to a stirred solution of ethyl (4-bromophenoxy)-2-phenylpropanoate (2 mmol, 1 eq) in a mixture of toluene and water (21 mL, 20:1); after 1 h at RT, Pd(PPh_3_)_4_ (0.06 mmol, 0.03 eq) was added. The reaction mixture was refluxed for 8 h and then quenched with 1 N HCl and ethyl acetate (10 mL, 1:1). The suspension was filtered through a Celite pad to remove the catalyst, and the filtrate was concentrated to dryness to give a yellow oil which was chromatographed on a silica gel column (*n*-hexane/ethyl acetate, 90:10, as eluent), obtaining a white solid in 40% yield. ^1^H-NMR (500 MHz, CDCl_3_) δ (ppm): 1.17 (t, 3H, CH_3_, J = 7.3 Hz), 1.19 (s, 3H, CH_3_), 4.22 (q, 2H, CH_2_, J = 7.3 Hz), 6.90–6.95 and 7.21–7.71 (m, 14H, aromatics); ESI-HRMS (C_23_H_22_O_3_) *m/z* (%) positive [M+Na]^+^: calculated: 369.1461, found: 369.1463

#### 3.1.14. Preparation of (4-Phenylphenoxy)-, (4-Chlorophenoxy)- and (4-Bromophenoxy)-2 phenylpropanoic Acids (**5**, **7** and **11**)

2 N NaOH (40 mmol in 20 mL of H_2_O, 20 eq) was added to a solution of the ethyl 2-substituted-2-phenylpropanoates (2 mmol, 1 eq) in THF (20 mL). The resulting mixture was stirred for 24 h at room temperature. Then, the organic solvent was removed under reduced pressure, the aqueous residue was acidified with 6 N HCl and then extracted with ethyl acetate. The collected organic portions were washed with brine, dried over anhydrous Na_2_SO_4_, filtered and concentrated to dryness. The solid residues were recrystallized from *n*-hexane (**5, 7**) or CHCl_3_/*n*-hexane (**11**) affording the title compounds.

(4-Phenylphenoxy)-2-phenylpropanoic acid (**5**). Pale yellow solid; yield = 60%; m.p. = 134–136 °C; ^1^H-NMR (500 MHz, CDCl_3_) δ (ppm): 1.97 (s, 3H, CH_3_), 6.86–6.92, 7.28–7.53 and 7.63–7.66 (m, 15H, 14H aromatics + COOH); ESI-HRMS (C_21_H_18_O_3_) *m/z* (%) negative [M-H]^−^: calculated: 317.1183, found: 317.1180; anal.: calcd for C_21_H_18_O_3_: C 79.22 %, H 5.70 %, found: C 78.78 %, H 5.68 %.

(4-Chlorophenoxy)- 2-phenylpropanoic acid (**7**). Yellow solid; yield = 27%; m.p. = 87–88 °C; ^1^H-NMR (300 MHz, CDCl_3_) δ (ppm): 1.90 (s, 3H, CH_3_), 6.48–6.77, 7.12–7.44 and 7.57–7.77 (m, 10H, 9H aromatics + COOH); GS-MS (methyl ester with diazomethane) *m/z* (%): 292 (2) [M+2]^+^, 290 (6) [M]^+^, 233 (9), 231 (28), 163 (94), 135 (88), 103 (100). ESI-HRMS (C_15_H_13_ClO_3_) *m/z* (%) negative [M-H]^−^: calculated: 275.0480, found: 275.0494.

(4-Bromophenoxy)-2-phenylpropanoic acid (**11**). Pale yellow solid; yield = 30%; m.p. = 114–117 °C; ^1^H-NMR (500 MHz, CDCl_3_) δ (ppm): 1.91 (s, 3H, CH_3_), 6.67–6.70, 7.26–7.42 and 7.56–7.59 (m, 10H, 9H aromatics + COOH); GS-MS (methyl ester with diazomethane) 332 (1) [M]^+^, 174 (40), 172 (42), 135 (47), 103 (100), 77 (28); anal.: calcd for C_15_H_13_BrO_3_: C 56.10 %, H 4.08 %, found: C 56.38 %, H 4.12 %.

#### 3.1.15. Synthesis of (*R*,*R*)- and (*S*,*R*)-Tetrahydro-4,4-dimethyl-2-oxofuran-3-yl-2-(4-bromophenoxy)-2-phenylpropanoates (**12a** and **12b**)

Dimethylaminopyridine (DMAP, 0.4 mmol, 0.2 eq) and 1-ethyl-3-(3-dimethylaminopropyl)carbodiimide (EDCI, 2.4 mmol, 1.2 eq), and (*R*)-pantolactone (6 mmol, 3 eq) were added to a stirred solution of the racemic acid **11** (2 mmol, 1 eq) in dichloromethane (20 mL). The reaction mixture was stirred at room temperature for 24 h, and afterwards the organic phase was evaporated to dryness, dissolved in ethyl acetate, and washed twice with 1 N HCl, NaHCO_3_ and brine. The organic layer was dried over Na_2_SO_4_ and evaporated to dryness to afford a yellow oil. The desired diastereomeric esters were obtained, as pale-yellow oils, by column chromatography on silica gel using *n*-hexane/ethyl acetate 70:30 as eluent. The two enriched fractions were further purified by a fractional crystallization from *n*-hexane obtaining the pure diastereomers *R*,*R* (**12a**) and *S*,*R* (**12b**) as white solids. Yield = 44%.

(*R*,*R*)-Tetrahydro-4,4-dimethyl-2-oxofuran-3-yl-2-(4-bromophenoxy)-2-phenylpropanoate (12a). ^1^H-NMR (500MHz, CDCl_3_): δ 0.81(s, 3H, CH_3_), 0.93 (s, 3H, CH_3_), 1.91 (s, 3H, CH_3_), 3.96 (s, 2H, CH_2_), 5.35 (s, 1H, CH), 6.82–6.86 (m, 2H, aromatics), 7.26–7.40 (m, 5H, aromatics), 7.63–7.67 (m, 2H, aromatics).

(*S*,*R*)-Tetrahydro-4,4-dimethyl-2-oxofuran-3-yl-2-(4-bromophenoxy)-2-phenylpropanoate (12b). ^1^H-NMR (500MHz, CDCl_3_): δ 0.74 (s, 3H, CH_3_), 0.99 (s, 3H, CH_3_), 2.00 (s, 3H, CH_3_), 3.90 (s, 2H, CH_2_), 5.35 (s, 1H, CH), 6.70–6.76 (m, 2H, aromatics), 7.26–7.40 (m, 5H, aromatics), 7.60–7.64 (m, 2H, aromatics).

#### 3.1.16. Synthesis of (*R*,*R*)- and (*S*,*R*)-Tetrahydro-4,4-dimethyl-2-oxofuran-3-yl 2-(4-phenylphenoxy)-2-phenylpropanoate (**13a** and **13b**)

Phenylboronic acid (4 mmol, 2 eq) and Cs_2_CO_3_ (3 mmol, 1.5 eq) were added, under a N_2_ atmosphere, to a stirred solution of ethyl (4-bromophenoxy)-2-phenylpropanoate (2 mmol, 1 eq) in a mixture of toluene and water (21 mL, 20:1); after 1 h at RT, Pd(PPh_3_)_4_ (0.03 eq) was added. The reaction mixture was refluxed for 6 h and then quenched with 1 N HCl and ethyl acetate (10 mL, 1:1). The suspension was filtered through a Celite pad to remove the catalyst, and the filtrate was concentrated under reduced pressure to give a yellow oil which was dissolved in ethyl acetate. The organic solution was then washed with a saturated aqueous solution of NaHCO_3_ and brine, dried over anhydrous Na_2_SO_4_, filtered and concentrated to dryness. The crude was chromatographed on a silica gel column (*n*-hexane/dichloromethane, 50:50, as eluent), obtaining a white solid.

(*R*,*R*)-Tetrahydro-4,4-dimethyl-2-oxofuran-3-yl-2-(4-phenylphenoxy)-2-phenylpropanoate (**13a**).

63% yield. ^1^H-NMR (500 MHz, CDCl_3_) δ (ppm): δ 0.80 (s, 3H, CH_3_), 0.90 (s, 3H, CH_3_), 1.97 (s, 3H, CH_3_), 3.95 (s, 2H, CH_2_), 5.37 (s, 1H, CH), 6.96–7.08 (m, 2H, aromatics), 7.29–7.53 (m, 10H, aromatics), 7.68–7.79 (m, 2H, aromatics); ESI-HRMS (C_27_H_26_O_5_) *m/z* (%) positive [M+Na]^+^: calculated: 453.1672, found: 453.1671.

(*S*,*R*)-Tetrahydro-4,4-dimethyl-2-oxofuran-3-yl-2-(4-phenylphenoxy)-2-phenylpropanoate (13b). 51% yield. ^1^H-NMR (500 MHz, CDCl_3_) δ (ppm): δ 0.74 (s, 3H, CH_3_), 0.98 (s, 3H, CH_3_), 1.56 (s, 3H, CH_3_), 3.94 (s, 2H, CH_2_), 5.37 (s, 1H, CH), 6.90–6.97 (m, 2H, aromatics), 7.29–7.53 (m, 10H, aromatics); 7.65–7.73 (m, 2H, aromatics).

#### 3.1.17. Preparation of (*R*)- and (*S*)-(4-Phenylphenoxy)-, (4-bromophenoxy)-2 phenylpropanoic acids (***R*-5**, ***S*-5** and ***R*-11**, ***S*-11**)

2.5 N NaOH (80 mmol in 32 mL of H_2_O, 40 eq) was added to a solution of the ethyl 2-substituted-2-phenylpropanoates (2 mmol, 1 eq) in 2-propanol (30 mL). The resulting mixture was stirred overnight at 65 °C. Then, the organic solvent was removed under reduced pressure, the aqueous residue was acidified with 6 N HCl and then extracted with ethyl acetate. The collected organic portions were washed with brine, dried over anhydrous Na_2_SO_4_, filtered and concentrated to dryness. The solid residues were recrystallized from *n*-hexane (**11**) or CHCl_3_/*n*-hexane (**5**) affording the title compounds.

(*R*)-(4-Phenylphenoxy)-2-phenylpropanoic acid (***R*-5**). White solid; yield = 54%; m.p. = 152–154 °C; [α]_D_ = −56 (c 1.0, MeOH); ESI-HRMS (C_21_H_18_O_3_) *m/z* (%) negative [M-H]^−^: calculated: 317.1183, found. 317.1175.

(*S*)-(4-Phenylphenoxy)-2-phenylpropanoic acid (***S*-5**). White solid; yield = 45%; m.p. = 149–152 °C; [α]_D_ = +57 (c 1.0, MeOH); ESI-HRMS (C_21_H_18_O_3_) *m/z* (%) negative [M-H]^−^: calculated: 317.1183, found. 317.1170.

(*R*)-(4-Bromophenoxy)-2-phenylpropanoic acid (***R*-11**). White solid; yield = 61%; m.p. = 97–99 °C; [α]_D_ = −50 (c 1.0, MeOH); ESI-HRMS (C_15_H_13_BrO_3_) *m/z* (%) negative [M-H]^−^: calculated: 318.9975, found: 318.9972.

(*S*)-(4-Bromophenoxy)-2-phenylpropanoic acid (***S*-11**). White solid; yield = 42%; m.p. = 99–101 °C; [α]_D_= +50 (c 1.0, MeOH); ESI-HRMS (C_15_H_13_BrO_3_) *m/z* (%) negative [M-H]^−^: calculated: 318.9975, found: 318.9972.

(*S*)-Methyl-(4-bromophenoxy)-2-phenylpropanoate (***S*-14**). ***S*-11** (2 mmol) was dissolved in methanol (15 mL) and added with a catalytic amount of H_2_SO_4_, and the resulting mixture was refluxed for 0.5 h. Next, the solvent was concentrated under reduced pressure and the resulting oil was dissolved in ethyl acetate and washed twice with sodium bicarbonate aqueous solution and brine. The organic portion was dried over anhydrous Na_2_SO_4_, filtered, and concentrated to dryness, affording a solid crude, which was chromatographed on a silica gel column (using *n*-hexane/ethyl acetate, 80:20, as eluent), obtaining the title compound; yield 70%. ^1^H-NMR (300 MHz, CDCl_3_) δ (ppm): 1.88 (s, 3H, CH_3_), 3.73 (s, 3H, CH_3_), 6.70–6.74 (m, 2H, aromatics), 7.28–7.41 (m, 5H, aromatic), 7.56–7.61 (m, 2H, aromatics); GC-MS *m/z* (%): 336 (1), 334 (1), 174 (45), 172 (45), 163 (79), 103 (100), 135 (72).

(*S*)-Methyl-(4-phenoxy)-2-phenylpropanoate (*S*-15). *S*-14 (2 mmol, 1 eq) was dissolved in methanol (30 mL) and stirred at RT under a H_2_ atmosphere (1.7 atm) in the presence of Pd/C (4 mmol, 2 eq). After 1 h the suspension was filtered through a Celite pad to remove the catalyst, and the filtrate was concentrated under reduced pressure to give a dark solid which was chromatographed on a silica gel column using *n*-hexane/ethyl acetate 70:30 as eluent, affording the title compound as a white solid in 57% yield. m.p. = 56–58 °C; [α]_D_ = +10; (c = 0.75, CHCl_3_); ESI-HRMS (C_16_H_16_O_3_) *m/z* (%) negative [M+Na]^+^: calculated: 279.0992, found: 279.0995; ^1^H-NMR (500 MHz, CDCl_3_) δ (ppm): 1.89 (s, 3H, CH_3_), 3.72 (s, 3H, CH_3_), 6.83–6.88 (m, 2H, aromatics), 6.97–7.02 (m, 1H, aromatic), 7.21–7.40 (m, 5H, aromatics), 7.61–7.66 (m, 2H, aromatics).

### 3.2. PPAR Assay

Reference compounds, the cell culture medium and other reagents were purchased from Sigma-Aldrich (Milan, Italy).

The expression vectors bearing the chimeric receptor containing the yeast Gal4-DNA binding domain fused to the human PPARα- or PPARγ-LBD, and the reporter plasmid for these Gal4 chimeric receptors (pGal5TKpGL3), comprising five repeats of the Gal4 response elements upstream of a minimal thymidine kinase promoter adjacent to the coding sequence for luciferase, were described in a previous work [42].

A culture of the human hepatoblastoma cell line HepG2 (Interlab Cell Line Collection, Genoa, Italy) was conducted in minimum essential medium (MEM) containing 10% heat-inactivated fetal bovine serum, 100 U of penicillin G mL^−1^, and 100 μg of streptomycin sulfate mL^−1^ at 37 °C in a humidified atmosphere of 5% CO_2_. For transactivation assays, cells were seeded in a 24-well plate at a concentration of 10^5^ cells per well, and were transfected after 24 h with CAPHOS, a calcium phosphate method, according to the manufacturer’s guidelines. Cell transfection was performed using expression plasmids encoding the fusion protein Gal4−PPARα-LBD or Gal4−PPARγ-LBD (30 ng), pGal5TKpGL3 (100 ng), and pCMVβgal (250 ng). Following transfection, cells were incubated for 4 h, after which they underwent treatment with the indicated ligands in triplicate for 20 h. Cell extracts were subsequently analyzed for luciferase activity via luminometry (VICTOR^3^ V multilabel plate reader, PerkinElmer). Ortho-nitrophenyl-β-D-galactopyranoside was used to measure β-Galactosidase activity, following a previously described method [50]. All transfection experiments were performed at least twice.

### 3.3. FAAH Inhibition Assay

To assess the activity of our compounds as FAAH inhibitors, 96-well black flat-bottom microtiter NBS plates (COSTAR flat black) were used. The assay was conducted in a total volume of 200 µL, with different concentrations of each tested compound (in triplicate) being preincubated for 10 min at room temperature in an appropriate fluorometric assay buffer (tris-HCl 125 mM, Na_2_EDTA·2H_2_O 1 mM, pH = 9.0) also containing the enzyme (FAAH Human recombinant, Cayman Chemical, Ann Arbor, MI, USA), while the plate was being kept in orbital shaking. Following this, the substrate (7-amino-4-methyl-2H-1-benzopyran-2-one-5Z,8Z,11Z,14Z-eicosatetraen-amide, AMC-AA, 5 µM final concentration) was added, and the plate was incubated for 2 h at 37 °C in a TECAN infinite M1000Pro plate reader (Tecan, Männedorf, Switzerland), reading the signal from each well every 30 s (λex = 340 nm, λem = 450 nm) and thus expressing FAAH activity as relative fluorescence units (RFU). The percent inhibition for each tested compound was calculated using control wells lacking the inhibitor and blank wells lacking both inhibitor and enzyme. IC_50_ values were calculated using GraphPad Prism 5.0 (GraphPad Software, La Jolla, CA, USA) and are expressed as mean ± SEM of at least two independent measurements performed in triplicate.

### 3.4. AChE and BuChE Inhibition Assay

Ellman’s spectrophotometric assay, adapted to a 96-well plate procedure, was used according to a previously described modified protocol [44]. All reagents and enzymes were commercially obtained from Sigma-Aldrich, (Milan, Italy). All assays were carried out in clear, flat-bottom, 96-well plates (Greiner Bio-One GmbH, Frickenhausen, Germany) in duplicate. Absorbance measurements were carried out with a TECAN Infinite M1000 Pro multiplate reader. Inhibition values were calculated as the mean of three independent experiments using GraphPad Prism, and are expressed as mean ± SEM.

### 3.5. Inhibition of Aβ_40_ Aggregation

A previously described method for the spectrofluorimetric assays measuring ThT fluorescence in the presence of Aβ was used [47]. Co-incubation samples were prepared in 96-well black, non-binding microplates (Greiner Bio-One GmbH, Frickenhausen, Germany) by diluting Aβ_40_ (EZBiolab, Carmel, IN, USA) and inhibitors to a final concentration of 30 and 100 µM respectively in PBS (pH 7.4) containing 2% 1,1,1,3,3,3-hexafluoro-2-propanol (HFIP). After 2 h of incubation at 25 °C, 25 µM ThT solution was added and fluorescence was determined with a multi-plate reader Infinite M1000 Pro (Tecan, Cernusco S.N., Italy). Assays were carried out in triplicate and values are reported as mean ± SEM.

### 3.6. Molecular Dockings

The three-dimensional structure of both the enantiomeric form of **5** and **11** were assembled within the Maestro software package [51], the proper ionization was assigned with *fixpka* complement of QUACPAC [52], and then molecular skeletons minimized throughout 10,000 steps of Steepest Descent with Open Babel [53] using the Universal Force Field. X-ray structures of the enzyme-inhibitor complexes for PPARα (pdb code 2P54) [54], PPARγ (pdb code 3B3K) [55], FAAH (pdb code 4DO3) [24], and AChE (pdb code 6O4W) [56] were selected as targets for dockings, and thus prepared with the Protein Preparation Wizard interface of Maestro removing the ligand and water molecules, adding hydrogen atoms by optimizing their position, and assigning the ionization states of acid and basic residues according to PROPKA prediction at pH 7.0. Electrostatic charges for protein atoms were loaded according to the AMBER UNITED force field [57], while the *molcharge* complement of QUACPAC [52] was used in order to achieve Marsili-Gasteiger charges for the inhibitors. Affinity maps for each enzyme were first calculated on a 0.375 Å spaced 80 × 80 × 80 (PPARα and PPARγ) and 80 × 80 × 80 (FAAH and AChE) Å^3^ cubic box, having the barycentre on the co-crystallized inhibitors poses, and the of the binding site available space was tested throughout 1000 runs of Lamarckian Genetic Algorithm (LGA) implemented in AUTODOCK 4.2.6 [58] using the GPU-OpenCL algorithm version [59]. The hydration force field parameters [60] were set in order to explicitly evaluate the contribution of water molecules in the binding, and the population size and the number of energy evaluations figures were set to 300 and 10,000,000, respectively.

### 3.7. Statistical Analyses

Statistical analyses were performed via one-way analysis of variance with Dunnet or Bonferroni post-test analysis for multiple group comparisons using GraphPad Prism version 5.0 (GraphPad Software, San Diego, CA, USA). Differences with *p* values of ≤0.05 were considered statistically significant.

## 4. Conclusions

This study demonstrates that some compounds of a new series of aryloxyacetic acid derivatives are able to exhibit high activity as PPARα and PPARγ agonists, moderate activity as FAAH and AChE inhibitors, and even some activity, although at high concentration, against Aβ peptide aggregation. In particular, compound **(*S*)-5** behaves as a potent PPARα full agonist (EC_50_ = 0.126 ± 0.011 μM, Emax = 86 ± 4) and potent PPARγ partial agonist (EC_50_ = 1.54 ± 0.24 μM, Emax = 38.7 ± 3.4) with a concomitant good activity on FAAH, which is the best of the whole series (IC_50_ = 5.3 ± 2.0 μM); in turn, **6** behaves as a potent PPARα “superagonist” (EC_50_ = 0.20 ± 0.03 μM, Emax = 129 ± 12) and potent PPARγ full agonist (EC_50_ = 0.88 ± 0.11 μM, Emax = 91 ± 12), whereas its inhibition activity on FAAH results approximately three-fold lower (IC_50_ = 14.8 ± 0.4 μM) than **(*S*)-5**. Both compounds also exhibit an intriguing moderate activity towards AChE, resulting in 37 ± 5 and 44 ± 4 percent of inhibition at 10 μM, respectively. Therefore, these compounds seem to offer, as also predicted from our modeling studies, the best opportunities for the investigation of the chemical modifications needed to achieve the appropriate simultaneous multi-target activity towards all four targets. As far as we know, these are the first examples of molecules endowed with this pharmacological profile, paving the way to a promising, yet unexplored, line of research for the identification of novel candidate drugs for the treatment of Alzheimer’s disease.

## Figures and Tables

**Table 1:** Docking results for selected targets.

Compound	Target	FEB ^(a)^	ΔE ^(b)^	EFF ^(c)^	POP ^(d)^
**(*S*)-5**	PPARα	−8.52	0.00	−0.355	66/1000
**(*R*)-5**		−9.56	0.00	−0.370	16/1000
**(*S*)-11**		−7.44	0.65	−0.392	28/1000
**(*R*)-11**		−9.21	0.00	−0.485	310/1000
**Wy-14,643**		−10.08	0.00	−0.480	194/1000
**(*S*)-5**	PPARγ	−10.67	0.00	−0.445	643/1000
**(*R*)-5**		−10.03	0.00	−0.418	96/1000
**(*S*)-11**		−10.37	0.00	−0.546	715/1000
**(*R*)-11**		−9.21	0.00	−0.485	231/1000
**rosiglitazone**		−9.41	0.81	−0.376	125/1000
**(*S*)-5**	FAAH	−8.30	0.41	−0.346	62/1000
**(*R*)-5**		−9.08	0.00	−0.378	590/1000
**(*S*)-11**		−7.41	0.42	−0.390	167/1000
**(*R*)-11**		−8.06	0.48	−0.424	134/1000
**JZL195**		−11.24	0.00	−0.351	44/1000
**(*S*)-5**	AChE	−9.54	0.00	−0.498	350/1000
**(*R*)-5**		−9.58	0.00	−0.399	540/1000
**(*S*)-11**		−8.47	0.00	−0.446	206/1000
**(*R*)-11**		−8.54	0.00	−0.449	612/1000
**donepezil**		−10.30	0.10	−0.368	508/1000

(^a^) Free Energy of Binding; (^b^) Energy difference between the selected pose and the relative global minimum; (^c^) Ligand efficacy; (^d^) Cluster members population.

**Table 2:** Biological properties of compounds **1**–**11**: PPARα/γ agonist activity and FAAH inhibition activity.

Compound	PPARα		PPARγ		FAAH
	EC_50_ (µM)	Emax ^a^ (%)	EC_50_ (µM)	Emax ^a^ (%)	IC_50_ (µM)
**1**	0.19 ± 0.04	116 ± 4	0.55 ± 0.12	62 ± 7	>50
**2**	1.75 ± 0.12	57 ± 4	0.72 ± 0.27	50 ± 1	24 ± 2.5
**3**	2.4 ± 0.4	69 ± 5	14.6 ± 3.5	9.6 ± 1.7	7.6 ± 0.9
**4**	4.7 ± 2.7	108 ± 12	13 ± 1.1	23 ± 9	6.9 ± 0.5
**5**	0.46 ± 0.04	105 ± 10	2.5 ± 0.6	39 ± 7	n.t.
**(** ***S*** **)-5**	0.126 ± 0.011	86 ± 4	1.54 ± 0.24	38.7 ± 3.4	5.3 ± 2.0
**(** ***R*** **)-5**	-	i	7.9 ± 2.5	14 ± 3	6.0 ± 0.8
**6**	0.20 ± 0.03	129 ± 12	0.88 ± 0.11	91 ± 12	14.8 ± 0.4
**7**	1.99 ± 0.23	63 ± 1	20.2 ± 1.2	22.5 ± 0.4	n.t.
**8**	1.57 ± 0.42	92 ± 6	5.06 ± 1.12	64 ± 1	n.t.
**9**	0.86 ± 0.03	86 ± 6	11.4 ± 1.8	18 ± 1	n.t.
**10**	7.9 ± 2.1	92 ± 22	-	8.1 ± 1.6	8.8 ± 0.5
**11**	0.73 ± 0.12	97 ± 1	13.8 ± 3.8	21 ± 2	n.t.
**(** ***S*** **)-11**	0.233 ± 0.034	77 ± 4	3.9 ± 0.5	40 ± 1.3	10.0 ± 1.8
**(** ***R*** **)-11**			22 ± 4	15.1 ± 2.5	6.8 ± 1.9
**Wy-14,643**	1.56 ± 0.30	100 ± 10			
**Rosiglitazone**			0.039 ± 0.003	100 ± 9	
**JZL195**					0.019 ± 0.003

^a^ Efficacy values were calculated as a percentage of the maximum obtained fold induction with the reference compounds; i = inactive at tested concentration; n.t. = not tested.

**Table 3:** Biological properties of compounds **3**–**11**: inhibition of AChE and self-mediated Aβ_40_ aggregation.

Compound	AChE	Aβ40 Aggr.
	i% @10 µM ^a^	i% @100 µM ^a^
**3**	39 ± 5	29 ± 4
**4**	49 ± 1	25 ± 5
**5**	n.t.	n.t.
**(*S*)-5**	37 ± 5	37 ± 5
**(*R*)-5**	37 ± 3	39 ± 3
**6**	44 ± 4	17 ± 2
**7**	n.t.	n.t.
**8**	n.t.	n.t.
**9**	n.t.	n.t.
**10**	35 ± 1	25 ± 6
**11**	n.t.	n.t.
**(*S*)-11**	47 ± 3	35 ± 4
**(*R*)-11**	38 ± 4	44 ± 4
**Donepezil**	0.017 ± 0.002 ^b^	14 ± 7 ^c^

^a^ Percent of inhibition at the reported concentration; ^b^ this value corresponds to the IC_50_ of donepezil; ^c^ percent of inhibition at 10 μM [46]; n.t.: not tested.

## Data Availability

Not applicable.

## Supplementary Materials

The following are available online, Figure S1: binding mode of the enantiomers of 5 and 11 and reference compounds to the selected targets.

## Sample Availability

Samples of the compounds are not vailable from the authors.
