# Supplementary material for: A New Series of Aryloxyacetic Acids Endowed with Multi-Target Activity towards Peroxisome Proliferator-Activated Receptors (PPARs), Fatty Acid Amide Hydrolase (FAAH), and Acetylcholinesterase (AChE)
Source: Molecules. 2022 Jan 31;27(3):958. doi: 10.3390/molecules27030958 (PMC8839882; doi:10.3390/molecules27030958)
Supplement: Supplementary file 1 [file molecules-27-00958-s001.zip › molecules-1537508-supplementary.pdf]

Supporting Information

A New Series of Aryloxyacetic Acids Endowed with Multi-Target Activity Towards Peroxisome Proliferator-Activated Receptors (PPARs), Fatty Acid Amide Hydrolase (FAAH), and Acetylcholinesterase (AChE)

Rosalba Leuci, Leonardo Brunetti, Antonio Laghezza, Luca Piemontese, Antonio Carrieri, Leonardo Pisani, Paolo Tortorella, Marco Catto, Fulvio Loiodice*

Dipartimento di Farmacia-Scienze del Farmaco, Università degli Studi di Bari "Aldo Moro", Via E. Orabona 4, 70125 Bari, Italy

* *Correspondence: fulvio.loiodice@uniba.it; Tel.: +39-080-5442778*

Table of Contents

Figure S1. Binding mode of the enantiomers of **5** and **11** and references to the selected targets. S2

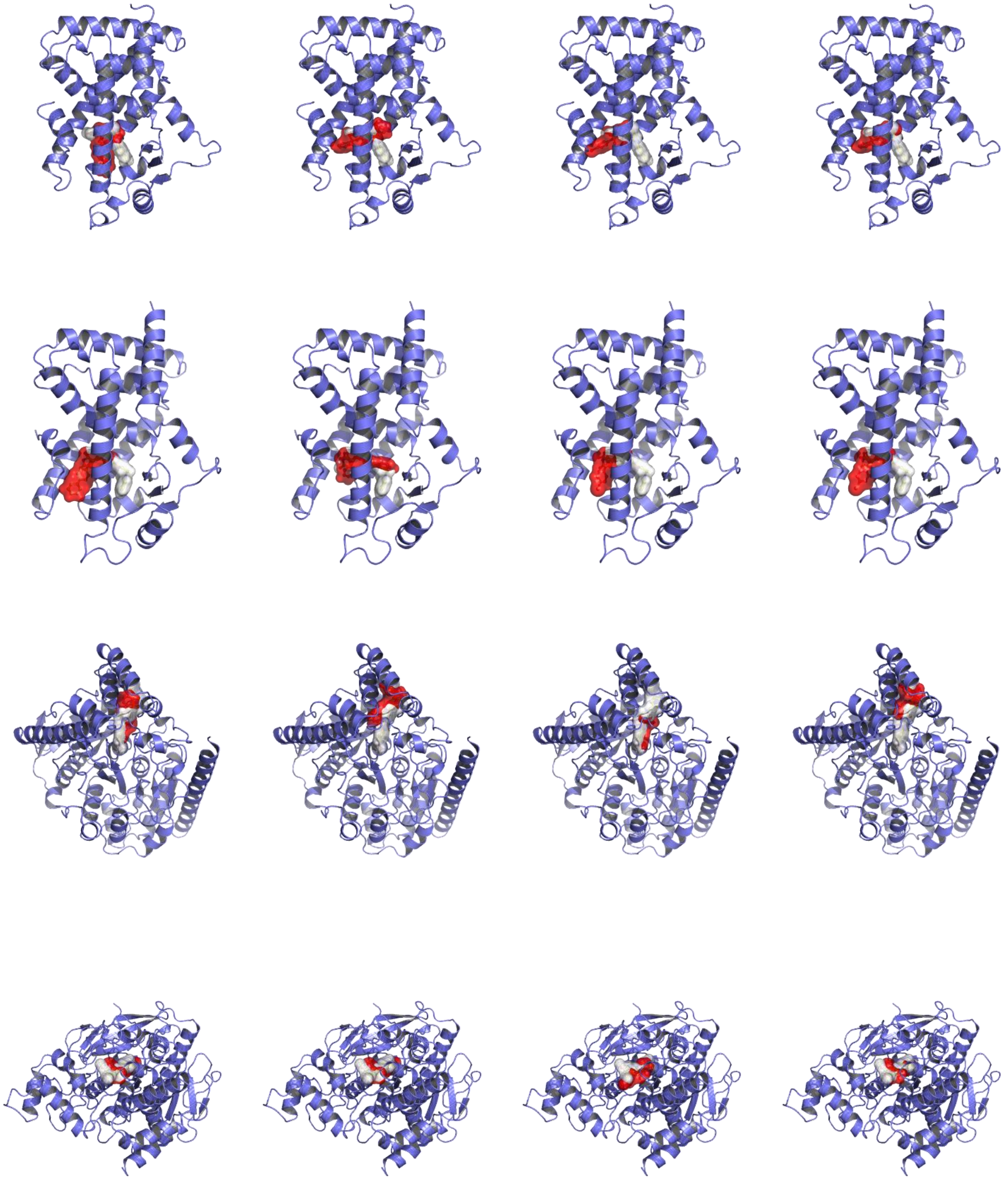

(*S*)-5 **(*R*)-5** **(*S*)-11** **(*R*)-11**

Figure S1. Full view of the binding mode to the selected targets (bottom to top: PPARα, PPARγ, FAAH, AChE). The (*S*) and (*R*)-enantiomers of **5** and **11** and reference compounds (Wy-14,643, rosiglitazone, JZL195, donepezil) are depicted as red and white surface, respectively.
